# The Diverse Spirit: Spiritual Care Perspectives and the Implications for Older Adults in Various Settings

**DOI:** 10.1093/geroni/igab046.506

**Published:** 2021-12-17

**Authors:** Lydia Manning, Chad Federwitz, Julie Hicks Patrick

## Abstract

Religiosity and spirituality are commonly supported and viewed as essential elements of well-being in old and very old age, particularly at end of life. These essential elements often include the exploration of the meaning in life, inner peace, belonging, contentment, and near-end-of-life-completion. The positive outcomes of religious and spiritual beliefs and practices have been well established. However, these experiences and related positive outcomes may not always include a diverse array of older adults. The spiritual care of older adults is becoming more culturally diverse and includes differing perspectives on what constitutes spiritual care, both in approach and practice. This symposium will explore the current state of spiritual care for older adults through a lens of cultural diversity and inter-religious/spiritual perspectives. A focus on the current practices of spiritual care for older adults and future implications will also be considered. Recommendations pertaining to future gerontological inquiry in the importance of spiritual care, as well as diverse approaches within gerontological practice will be highlighted and discussed.

